# Matrine Attenuates *Streptococcus agalactiae* Virulence by Suppressing Capsular Polysaccharide Synthesis and Host Adhesion Pathways

**DOI:** 10.3390/microorganisms13061192

**Published:** 2025-05-23

**Authors:** Shijiao Guo, Kaiming Wang, Hua Zhang, Chaochao Luo, Zixuan Zhao, Jinjin Tong

**Affiliations:** 1 Animal Science and Technology College, Beijing University of Agriculture, Beijing 102206, China; chinaguoshijiao@163.com (S.G.); 202120311292@bua.edu.cn (Z.Z.); 2 College of Veterinary Medicine, Beijing University of Agriculture, Beijing 102206, China; 202330311008@bua.edu.cn (K.W.); huazhang0914@163.com (H.Z.); 3 College of Life Sciences, Shihezi University, Shihezi 832003, China; luochaochao839505@163.com

**Keywords:** matrine, *Streptococcus agalactiae*, capsule formation, biofilm formation, transcriptomics

## Abstract

*Streptococcus agalactiae* (GBS) is a major pathogen causing mastitis in dairy cows while causing oxidative stress. Matrine is an alkaloid compound extracted from the roots of *Sophora flavescens*, a plant used in traditional Chinese medicine. It possesses antioxidant, immunomodulatory, anti-inflammatory, and pro-apoptotic properties. The aim of this study was to investigate the regulatory effects of matrine on the virulence of the ATCC strain (ATCC13813) and clinical GBS strains by transcriptome analysis and qRT-PCR validation. The results showed that the ABC transporter, peptidoglycan biosynthesis, and quorum-sensing pathways were significantly altered in ATCC (4 mg/mL) and GBS (12 mg/mL) strains after matrine treatment at MIC concentrations. Additionally, genes related to invasion and immune escape, including *CylE*, *CAMP*, *ScpB*, and *CpsA*, and genes related to the expression of adhesion and virulence factors, such as *Bac*, *Lmb*, *PI2a*, and *PI2b*, were significantly downregulated (*p* < 0.05). Overall, these data suggest that matrine effectively inhibits the virulence genes of GBS, thereby reducing immune evasion and infection by decreasing the synthesis of capsular polysaccharides and host cell adhesion.

## 1. Introduction

Mastitis remains a significant challenge in dairy farming, impacting animal welfare, milk quality, and global food safety [1]. *Streptococcus agalactiae* (GBS) is a major pathogen responsible for high infection rates, reduced milk production, and economic losses [2]. The administration of antibiotics, particularly via intramammary infusion, is associated with prolonged residue accumulation in milk, necessitating extended withdrawal periods, which not only impose substantial economic burdens on dairy producers due to product discard but also exacerbate the global challenge of antimicrobial resistance (AMR) through environmental dissemination and selective pressure on bacterial populations [3]. Given rising consumer demand for high-quality, safe dairy products, GBS is not only a major cause of mastitis in dairy cows but also a significant pathogen in human neonates and immunocompromised individuals. Vertical transmission from maternal genital flora to neonates can result in severe infections, including sepsis and meningitis, which are often difficult to diagnose in the early stages [4]. Due to its widespread impact, GBS infection should be considered not merely a veterinary issue but a broader public health concern. Therefore, developing effective natural compounds to control GBS-induced mastitis is crucial for ensuring the sustainability of dairy production and safeguarding both animal and human health.

Matrine is a natural alkaloid mainly extracted from the roots of *Sophora flavescens* [5], a traditional Chinese medicinal herb. This plant-derived compound exhibits a broad range of pharmacological activities, including antioxidant, immunomodulatory, and anti-inflammatory effects. Our previous study demonstrated that a 7-day intramammary infusion of matrine–chitosan hydrogel (MCH) effectively reduced somatic cell counts, modulated milk microbiota composition, and altered metabolite profiles in cows with subclinical mastitis, suggesting that matrine exerts antibacterial and anti-inflammatory effects, potentially via regulation of glycerophospholipid and sphingolipid metabolic pathways [6]. In addition, studies have shown that matrine has a broad spectrum of antimicrobial effects, including its ability to significantly inhibit the adhesion of *Staphylococcus aureus* (*S*. *aureus*), a key step in bacterial pathogenesis [7]. Meanwhile, Zhou et al. developed a konjac glucan/fish gelatin hydrogel loaded with matrine, which showed enhanced blood compatibility and strong antibacterial activity against *Escherichia coli* and *S*. *aureus*, demonstrating the antibacterial function of matrine [8]. However, despite its known activity against pathogens like *S*. *aureus* and *Escherichia coli*, its mechanism of action against GBS remains unexplored.

During infection, GBS employs various strategies to evade host immune detection, allowing for successful colonization and persistence. One key mechanism involves modifying its surface molecules to mimic host structures. It has been reported that sialic acid within the GBS capsule prevents immune recognition by disguising the bacterium as self, thereby evading host defenses [9]. Similarly, O-acetylation of capsular polysaccharide sialic acid further enhances immune evasion, complicating host immune responses [10]. Beyond the capsule, biofilm formation represents another pivotal virulence strategy. GBS utilizes surface adhesins (e.g., *BsaB*, *FbsA*) and extracellular matrix components to establish structured biofilms on host tissues [11]. These biofilms not only promote antibiotic resistance by limiting drug penetration but also shield bacteria from phagocytic clearance through physical barrier formation [12]. In addition to immune evasion, GBS colonization and proliferation in mammary epithelial cells depend on its ability to adhere to host tissues. Bacterial adhesion is critical for colonization, facilitating intracellular persistence and infection progression [10]. The increasing prevalence of drug-resistant GBS strains poses a major challenge to infection control, with significant implications for veterinary medicine, public health, and dairy production [13].

Given these findings, we hypothesize that matrine may inhibit *Streptococcus agalactiae* (*S. agalactiae*) adhesion and colonization by preventing biofilm formation and interfering with key virulence pathways. The primary objective of this study was to evaluate the anti-virulence effects of matrine against *S. agalactiae*. These findings highlight the potential of matrine as a promising natural alternative to antibiotics for the prevention and control of GBS-related mastitis in the dairy industry.

## 2. Materials and Methods

### 2.1. Cell Culture and Bacteria Growth

The Bovine mammary epithelial cells (BMECs) were obtained from Beijing University of Agriculture, Beijing, China, and were cultured in Dulbecco’s modified Eagle medium (DMEM)/F-12 (Gibco, Waltham, MA, USA) supplemented with 20% (*v*/*v*) fetal bovine serum, 5 µg/mL insulin (Sigma-Aldrich, St. Louis, MO, USA), 1 µg/mL hydrocortisone, 1 µg/mL corporin (Sigma-Aldrich, St. Louis, MO, USA), 5 µg/mL transferrin (Sigma-Aldrich, St. Louis, MO, USA), and antibiotics (100 µg/mL gentamicin and 100 µg/mL penicillin-streptomycin; Hyclone, Logan, UT) under a 5% CO_2_ atmosphere. Animal experiments were conducted in compliance with the ethical guidelines of the Beijing University of Agriculture Institutional Animal Care and Use Committee (No. BUA812X08080, approved on 13 June 2021). Three clinical GBS strains (GBS 1, 2, 3) were isolated from milk samples of 68 randomly selected dairy cows diagnosed with mastitis (2.5 ± 0.5 parity). Clinical mastitis cases were defined as cows with persistent high SCC (>200,000 cells/mL) over two months. None of the cows had received antibiotic treatment in the 30 days prior to sampling. Mastitis was confirmed using the California Mastitis Test (CMT) at the Beijing Dairy Centre, Beijing, China. The isolated strains were identified as GBS through bacterial culture, biochemical tests, the *CAMP* test, PCR amplification of the *cfb* gene, and 16S rRNA sequencing, which confirmed >99% homology with sequences logged in NCBI, consistent with findings from our previous studies [14]. The standard strain ATCC 13813 (ATCC) was purchased from the American Type Culture Collection. The matrine stock solution was prepared by accurately weighing 19.2 g of matrine and dissolving it in sterile phosphate-buffered saline (PBS, pH 7.4) to obtain a final concentration of 50 mg/mL. For experimental use, matrine was also dissolved in bacterial culture medium at the required concentrations and sterilized prior to use. In all experiments, the control group received an equal volume of sterile saline without matrine to ensure consistent solvent exposure between treatment and control groups.

### 2.2. Drugs and Chemicals

Matrine (SM8130) was purchased from Beijing Solarbio Science and Technology Co., Ltd. (Beijing, China). DMEM/F-12 and fetal bovine serum (FBS) were purchased from Gibco. Brain–heart infusion (BHI) was purchased from OXOID (Basingstoke, UK). Phosphate-buffered saline (PBS) was purchased from Beijing Wobiesen Technology Co., Ltd. (Beijing, China). All chemicals were of analytical grade and used without purification.

### 2.3. Cell Viability Assay

The BMEC were seeded in 96-well plates (1 × 10^4^ per well), allowed to adhere overnight, and the supernatant was discarded. The cells were washed twice in PBS, and 100 µL of DMEM/F-12 was added for 12 h to synchronize the cells. Then, the cells were washed twice with PBS, and 100 µL of DMEM/F-12 containing matrine at final concentrations of 0 (control), 25, 50, 75, 100, 125, or 150 µg/mL was added and incubated at 37 °C for 24 h under 5% CO_2_. The cells were then washed twice in PBS, and 110 µL of DMEM/F-12 was added to each well, including 10 µL of cell counting kit (CCK)-8 reagent (Dojindo Laboratories, Kumamoto, Japan), and incubated at 37 °C for 3 h. Finally, the optical density at 450 nm was measured, and the survival rate was calculated according to kit instructions. A survival rate above 90% was the goal after drug treatment.

### 2.4. Determination of the Minimum Inhibitory Concentration

The minimum inhibitory concentration (MIC) of matrine against GBS was determined using a modified broth microdilution method based on CLSI M27-A3 guidelines [15]. Clinical isolates of GBS and the reference strain ATCC 13813 were cultured overnight at 37 °C in BHI broth. After reaching the exponential growth phase, bacterial suspensions were adjusted to an initial concentration of 10^5^ CFU/mL in BHI medium. A 100 μL aliquot of this suspension was then added to each well of a sterile 96-well microtiter plate containing 100 μL of matrine solutions at final concentrations of 2, 4, 6, 8, 10, and 12 mg/mL, prepared in BHI. Each concentration was tested in triplicate. Wells containing BHI medium only served as negative (blank) controls, while wells containing BHI with bacteria but without matrine served as positive growth controls. Plates were incubated at 37 °C for 24 h, and bacterial growth was assessed by measuring the optical density at 600 nm (OD_600_). The MIC was defined as the lowest concentration of matrine that resulted in complete inhibition of visible bacterial growth. Only results from tests with valid controls (i.e., proper growth in positive controls and no contamination in blanks) were considered for analysis.

### 2.5. Time-Dependent Killing Curve

To evaluate the time-dependent antibacterial activity of matrine, the ATCC strain was cultured to the exponential growth phase and diluted in fresh BHI medium to a final concentration of 1 × 10^5^ CFU/mL. The ATCC strain was treated with final matrine concentrations of 1, 2, 3, and 4 mg/mL, while the control group received an equivalent volume of sterile culture medium instead of matrine to maintain consistent experimental conditions. The cultures were incubated with agitation at 37 °C for 18 h. Samples were collected at predetermined time points (0, 2, 4, 8, 10, 12, and 24 h). The optical density at 600 nm was measured hourly using a spectrophotometer to generate growth curves. These curves reflected the inhibitory effect of matrine over time and were used to assess its bactericidal or bacteriostatic properties.

### 2.6. Evaluation of Cell Adhesion

BMECs were cultured in 24-well plates using DMEM medium and maintained in a humidified incubator at 37 °C with 5% CO_2_ for 24 h. GBS cultures, grown overnight in BHI medium, were washed and resuspended in antibiotic-free DMEM to achieve a concentration of 1 × 10^5^ CFU/mL, determined spectrophotometrically at 600 nm. The BMECs were then infected with GBS at a multiplicity of infection (MOI) of 50:1. After 3 h, the infected cells were washed three times with PBS and lysed using 0.1% Triton X-100. Bacterial lysates were serially diluted and plated on nutrient agar, followed by incubation at 37 °C overnight for CFU quantification. The adherence assay was repeated independently in triplicate.

### 2.7. Cow Whole-Blood Killing Assays

Blood samples were collected from the middle tail vein of cows using vacuum tubes prior to morning feeding, following procedures approved by Beijing Dairy Centre, Beijing, China. Anticoagulation was achieved with heparin. For the killing assay, 70 µL of whole blood was mixed with 20 µL of matrine at concentrations of 2, 4, 6, and 8 mg/mL. The control group received 20 µL of physiological saline, and all samples were combined with 10 µL of GBS suspension (1 × 10^5^ CFU). The mixture was incubated at 37 °C with gentle rotation for 2.5 h. After incubation, the samples were plated on BHI agar to determine GBS survival, expressed as a percentage of the initial inoculum.

### 2.8. TEM Assays

The ability of matrine to form polysaccharide capsules against *S. agalactiae* ATCC 13813 and GBS clinical strains was assessed by transmission electron microscopy. The cultured *S. agalactiae* ATCC 13813 and clinical GBS strain were each incubated with matrine at its MIC at 37 °C for 6 h. In the control group, matrine solution was replaced with an equal volume of PBS. At the end of the co-incubation, the samples were centrifuged, and the supernatant was discarded. After washing three times with PBS, 2.50% glutaraldehyde fixative was added and fixed overnight at 4 °C. The fixed samples were dehydrated step-by-step with gradient alcohol, such as 30%, 50%, 70%, 90%, and 100% [16]. Dehydration was followed by drying to remove water. A Hitachi H-7650 TEM (Hitachi, Tokyo, Japan) was used to observe the morphological and ultrastructural changes in GBS cells.

### 2.9. Biofilm Inhibition

The biofilm formation inhibition assay was performed to assess the preventive effect of matrine on biofilm formation, following a modified protocol from a previous study [17]. Briefly, clinical GBS (GBS1) cultures were grown in TSB medium for 16 h, adjusted to an optical density (OD_600_) corresponding to 1 × 10^5^ CFU/mL, and diluted 1:100 in fresh TSB supplemented with 1.0% glucose. The ATCC strain and GBS clinical strains were treated with matrine at concentrations of 4, 8, and 10 mg/mL, while the control group was supplemented with an equivalent volume of sterile culture medium to replace matrine. After 24 h of incubation, biofilms were stained using 0.1% crystal violet for 15 min. Unbound stain was removed by rinsing the wells three times with PBS. The biofilm biomass was quantified by solubilizing the crystal violet in 33% glacial acetic acid (200 μL) and measuring absorbance at 570 nm using a microplate reader (BioTek, Winooski, VT, USA). Biofilm biomass was calculated by the following equation:

$$\mathrm{Biofilm}\;\mathrm{Activity}\;(\%)=\left(\frac{{OD}_{Treated}}{{OD}_{Control}}\right)\times 100$$

### 2.10. Transcriptional Profiling and Analysis

Transcriptional profiling of GBS was conducted by the Biozeron Company in Shanghai, China (BZYWM20201116001). Total RNA was extracted from GBS cultures treated with or without 0.5 mM indole using TRIzol reagent (Invitrogen, Waltham, MA, USA). The quality and concentration of RNA were assessed using a Bioanalyzer 2100 (Agilent, Santa Clara, CA, USA) and NanoDrop 2000 (Thermo Fisher Scientific, Waltham, MA, USA), respectively. RNA libraries were prepared with the TruSeq RNA library preparation kit (Illumina, San Diego, CA, USA), and residual rRNA was removed using the RiboZero rRNA removal kit (Epicenter, Zeist, The Netherlands). Sequencing was performed on an Illumina Hiseq platform. Raw paired-end reads were processed using SeqPrep (https://github.com/jstjohn/SeqPrep, accessed on 1 May 2024) for trimming and Sickle (https://github.com/najoshi/sickle, accessed on 1 May 2024) for quality control. The cleaned reads were aligned to the reference genome using Rockhopper (http://cs.wellesley.edu/~btjaden/Rockhopper/, accessed on 1 January 2025). Differential gene expression was analyzed with EdgeR (https://bioconductor.org/packages/release/bioc/html/edgeR.html, accessed on 1 June 2024), identifying significant changes with a fold change ≥ 2 and a *p*-value < 0.05. Functional enrichment was performed using GO analysis with Goatools (https://github.com/tanghaibao/Goatools, accessed on 1 January 2025) and KEGG pathway analysis with KOBAS (http://bioinfo.org/kobas, accessed on 1 January 2025). These analyses provided insights into significant biological pathways and functions influenced by indole treatment.

### 2.11. Quantitative Real-Time PCR Analysis

The presence of virulence genes in GBS and the ATCC strain was verified using PCR with the primers listed in Supplementary Table S1. Quantitative real-time PCR (qRT-PCR) was employed to analyze the expression of these virulence genes. For qRT-PCR, GBS strains cultured in BHI medium (AT-0 or GB-0) for 24 h were used. Total RNA was isolated using the RNA isolator Total RNA Extraction Reagent (R401-01-AA, Vazyme, Nanjing, China) according to the manufacturer’s protocol. The obtained RNA was dissolved in diethylpyrocarbonate (DEPC)-treated water. The purity and concentration of RNA were determined with SpectraMax i3x (Molecular Devices, San Jose, CA, USA), and the 260/280 ratio was between 1.8 and 2.0. According to the manufacturer’s instructions, 1 μg of total RNA was reverse-transcribed to generate cDNA using the HiScript^®^ II Q RT SuperMix for qPCR (R223, Vazyme, Nanjing, China). The reactions were performed in 20 μL volumes with Advanced™ Universal SYBR^®^ Green Supermix on a MIC real-time PCR system(Bio-Rad, Hercules, CA, USA). Triplicate reactions were run under the following conditions: polymerase activation at 95 °C for 15 min, denaturation at 95 °C for 15 s, annealing at 60 °C for 20 s, and elongation at 72 °C for 20 s, repeated for 45 cycles. Relative mRNA expression was quantified using the 2^−ΔΔCt^ method, with 16S rRNA as the reference gene.

### 2.12. Statistical Analysis

The results are expressed as mean ± standard deviation (SD) based on at least three independent experiments. Statistical analyses were conducted using GraphPad Prism software (version 9), employing one-way ANOVA followed by Bonferroni’s post hoc test for multiple comparisons. Statistical significance is indicated by asterisks (* *p* < 0.05, ** *p* < 0.01).

## 3. Results

### 3.1. The Inhibition of GBS by Matrine in a Concentration-Dependent Manner

**Figure 1:**
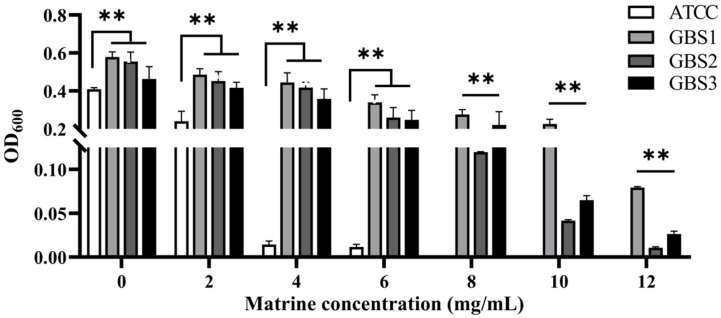
Minimum inhibitory concentration of matrine against *S. agalactiae*. ** indicates very significant difference (*p* < 0.01).

**Figure 2:**
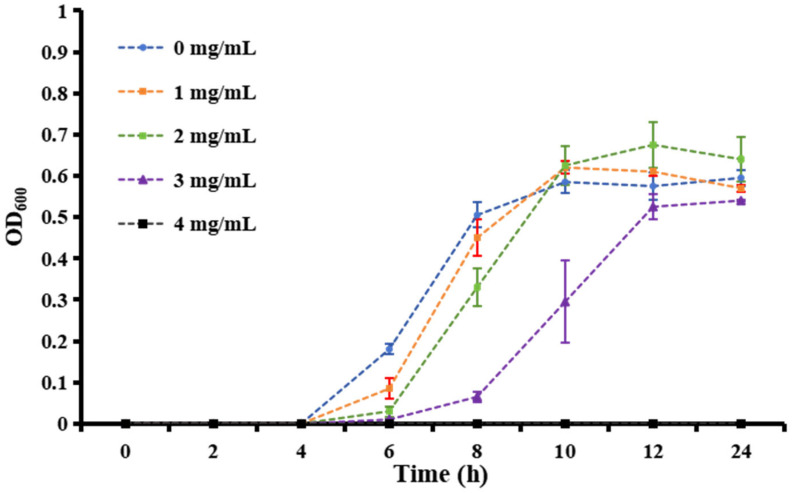
Growth curves of *S. agalactiae* in the presence of matrine at different concentrations from 0 to 5 mg/mL.

Over 600 milk samples from lactating Holstein cows with subclinical mastitis were analyzed using 16S amplicon sequencing to identify GBS infections. The antibacterial effect of matrine against GBS was assessed by determining its MIC, defined as the lowest concentration achieving 80% growth inhibition. As shown in Figure 1, the MIC values of clinically isolated strains were significantly higher than those of the ATCC strain (*p* < 0.01). After 12 h of co-cultivation, matrine at 2 mg/mL significantly inhibited the proliferation of the ATCC strain compared to the untreated group (*p* < 0.01). Similarly, clinically isolated GBS strains showed reduced proliferation at matrine concentrations between 2 and 4 mg/mL, with significant differences from the control group (*p* < 0.01). Moreover, when the matrine concentration exceeded 4 mg/mL, the ATCC strain was completely inhibited (*p* < 0.01) (Figure 1). We found that matrine had an MIC of 4 mg/mL against the ATCC strain, while the clinical isolates GBS1, 2, and 3 had mics of 12, 8, and 12 mg/mL, respectively, which is consistent with previous reports. Among the three clinical isolates, GBS1 exhibited the highest MIC (12 µg/mL) and the strongest biofilm formation capacity, suggesting a possible correlation between biofilm-forming ability and matrine tolerance. The antibacterial effect of matrine on GBS was further validated through a time-kill assay. As shown in Figure 2, time-kill curves demonstrated that matrine at concentrations ranging from 1 to 5 mg/mL significantly suppressed the growth of the ATCC strain within the first 6 h, with complete inhibition observed within the initial 4 h. These findings indicate that matrine exerts its inhibitory effects on GBS in a concentration-dependent manner.

### 3.2. Effect of Matrine on the Adhesion of GBS

**Figure 3:**
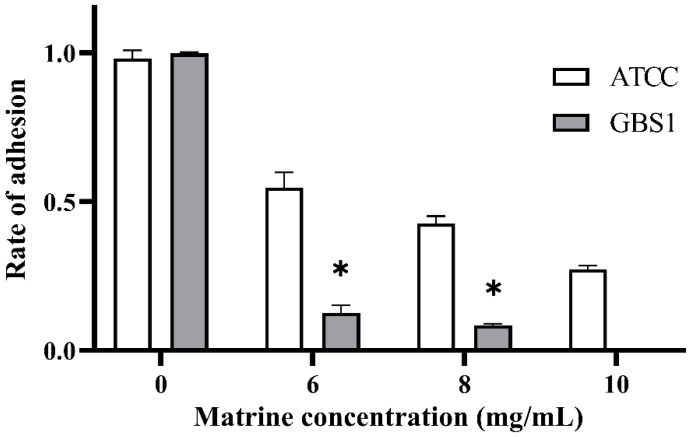
Effect of matrine on BMECs adhesion of *S. agalactiae*. * indicates significant difference (*p* < 0.05).

The effect of matrine on GBS adhesion was evaluated using an in vitro adhesion assay with BMECs. As shown in Figure 3, all GBS strains treated with matrine exhibited significantly lower adhesion rates to BMECs compared to untreated strains (*p* < 0.05). Notably, matrine had a more pronounced inhibitory effect on clinically isolated strains than on the ATCC strain. Upon treatment with 6 mg/mL matrine, the adhesion rate of the ATCC strain decreased to 58.62% (*p* < 0.05), whereas the adhesion rate of clinically isolated strains was significantly reduced to 14.6% (*p* < 0.05). Moreover, the adhesion rate of the ATCC strain gradually declined with increasing matrine concentration. These results indicate that matrine effectively reduces GBS adhesion to BMECs in a dose-dependent manner, with a stronger inhibitory effect on clinical isolates.

### 3.3. Effect of Matrine on Whole-Blood Viability of GBS

**Figure 4:**
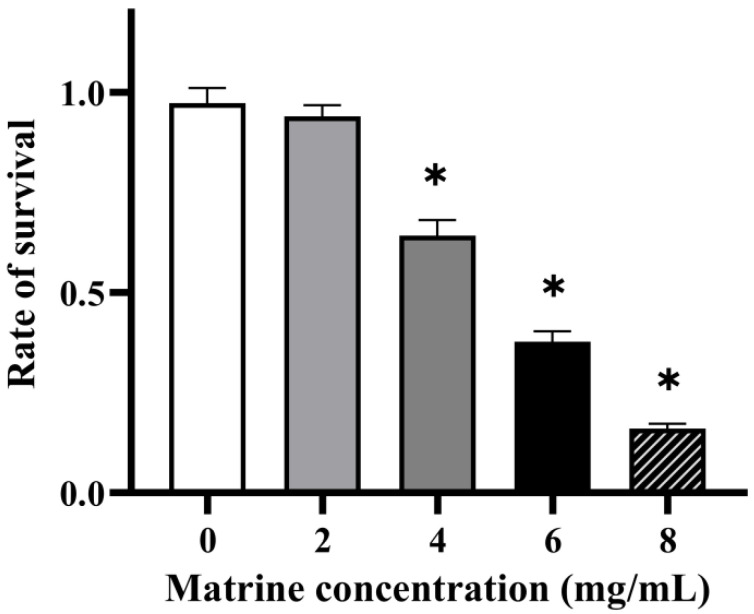
Effect of matrine on blood killing of *S. agalactiae.* * indicates significant difference (*p* < 0.05).

The ability of GBS to evade phagocytosis was assessed through a whole-blood survival assay, as neutrophils and other polymorphonuclear leukocytes (PMNs) play a crucial role in pathogen clearance. As shown in Figure 4, matrine treatment significantly reduced the survival rate of GBS in whole blood, indicating a decreased resistance to phagocytosis. At a concentration of 4 mg/mL, the survival rate of GBS decreased to 70.40% (*p* < 0.05), while 6 mg/mL matrine further reduced survival to 41.40% (*p* < 0.05). At 8 mg/mL, the survival rate declined sharply to 17.98% (*p* < 0.05). These results demonstrate a concentration-dependent effect, with increasing matrine levels leading to a progressive decline in GBS survival in whole blood.

### 3.4. Effect of Matrine on Capsular Polysaccharides of GBS

**Figure 5:**
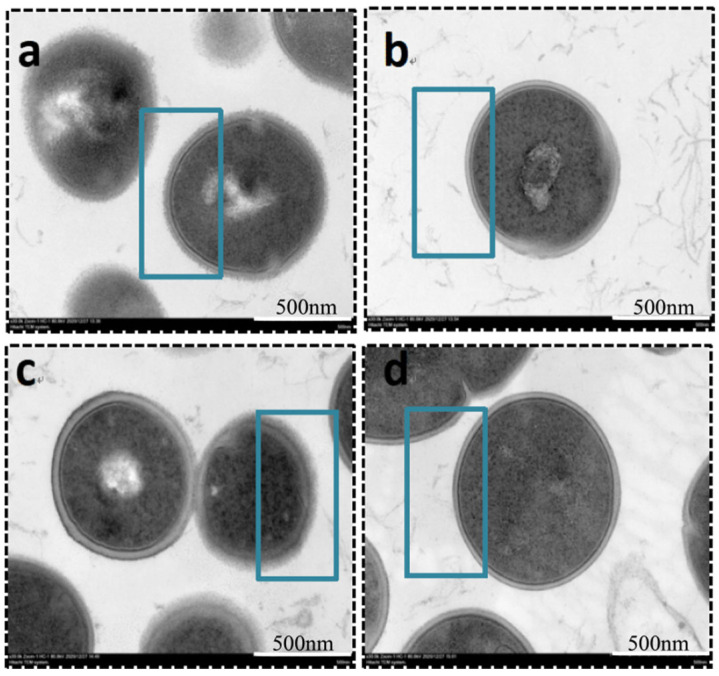
TEM images of *S. agalactiae* after incubation with matrine. (**a**) ATCC control; (**b**) ATCC MIC; (**c**) *GBS*1 control; (**d**) *GBS*1 MIC. Scale bar: 500 nm.

Many invasive microorganisms produce capsular exopolysaccharides, which serve as a protective barrier against phagocytosis. Transmission electron microscopy (TEM) analysis revealed that the polysaccharide capsules of both clinically isolated GBS and the ATCC strain were significantly inhibited following treatment with matrine at its MIC concentration (Figure 5). As indicated in Figure 5, untreated GBS exhibited a thick, well-defined polysaccharide capsule, whereas matrine-treated bacteria displayed a smooth cell surface with a complete loss of the capsule. Notably, matrine treatment did not affect bacterial morphology or cytoskeletal integrity, suggesting that its inhibitory effect specifically targets capsular formation without causing structural damage to the cells.

### 3.5. Effect of Matrine on Biofilm of GBS

**Figure 6:**
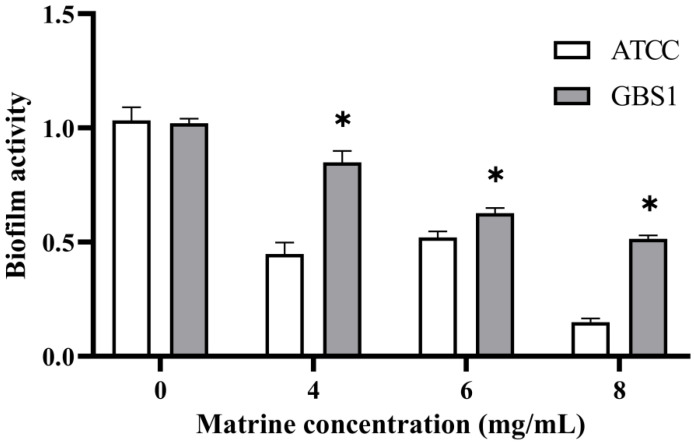
Effect of matrine on biofilm inhibition of *S. agalactiae*. * indicates significant difference (*p* < 0.05).

To further investigate the impact of matrine on GBS immune evasion and invasion, its effects on biofilm formation were assessed using a biofilm formation assay. As shown in Figure 6, matrine exhibited strong biofilm inhibitory activity against both the ATCC strain and GBS clinical strains. At matrine concentrations of 4, 6, and 8 mg/mL, biofilm activity in the ATCC strain was reduced to 44.06%, 46.18%, and 16.40%, respectively (*p* < 0.05). Similarly, biofilm formation in clinically isolated strains decreased to 83.52%, 66.00%, and 51.90% (*p* < 0.05) at the same concentrations. These results indicate that matrine effectively disrupts GBS biofilm formation in a dose-dependent manner, thereby potentially impairing its ability to evade host immune responses.

### 3.6. Mechanisms by Which Matrine Regulates the Expression of GBS-Associated Virulence Genes

**Figure 7:**
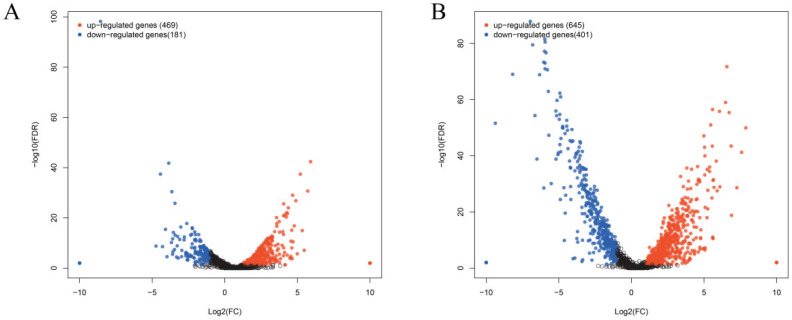
Effects of matrine on transcription of *S. agalactiae*. Each point shows a changed gene, and the genes that are significantly upregulated and downregulated are expressed in red and blue, respectively. Black indicates genes that are neither up-regulated nor down-regulated. The transverse coordinate in the image is the multiple of the change in *Streptococcus agalactiae* gene expression before and after the use of matrine. (**A**) ATCC; (**B**) *GBS*1.

**Figure 8:**
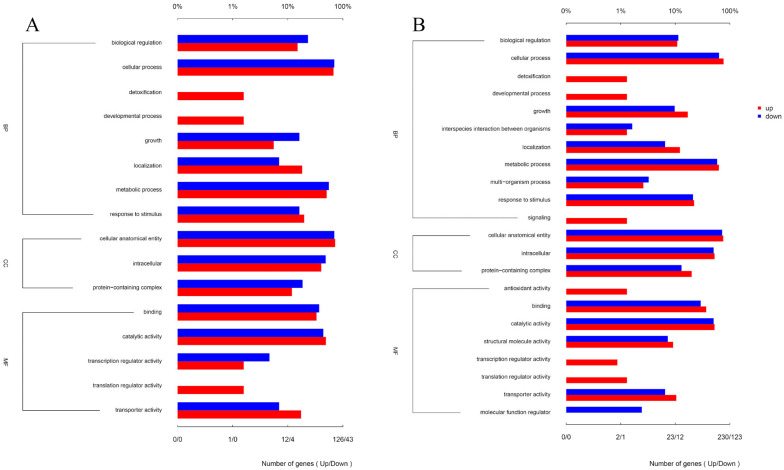
Effects of matrine on GO annotation of genes of *S. agalactiae.* (**A**) ATCC; (**B**) *GBS*1.

Matrine reduces the invasion and immune evasion capabilities of GBS by decreasing adhesion rates, inhibiting capsular polysaccharide production, and suppressing biofilm formation. To further investigate whether matrine modulates GBS gene expression, transcriptional profiling was performed. After co-cultivation with matrine at 4 mg/mL, significant changes in gene expression were observed. In the ATCC strain, 650 genes were differentially expressed, with 469 upregulated and 181 downregulated. In clinical isolates, 1046 genes exhibited significant changes, including 645 upregulated and 401 downregulated genes (Figure 7). Gene Ontology (GO) functional annotation classified these genes into three major categories: Biological Process (BP), Cellular Component (CC), and Molecular Function (MF). GO enrichment analysis identified the top 20 signaling pathways influenced by matrine, as shown in Figure 8. Notably, key biological processes related to virulence factor expression and secretion, such as glycosaminoglycan degradation, peptidoglycan biosynthesis, ABC transporters, two-component systems, quorum sensing, biofilm formation, bacterial chemotaxis, protein export, and the bacterial secretion system, were significantly altered in both the ATCC strain and GBS clinical strains (Figure 8).

**Figure 9:**
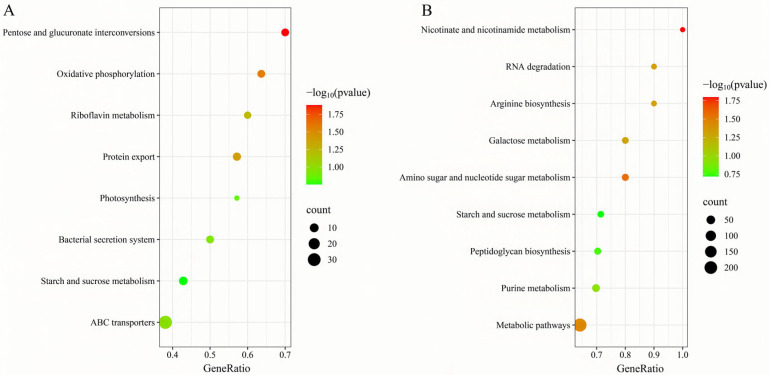
Effects of matrine on KEGG enrichment of genes of *S. agalactiae*. (**A**) ATCC; (**B**) *GBS*1.

**Figure 10:**
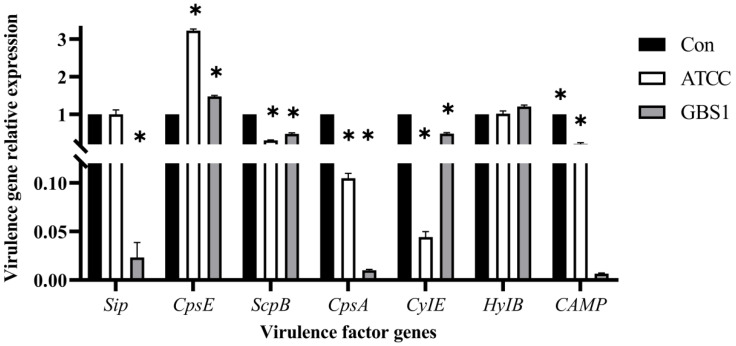
Effects of matrine on invasion and immune escape genes of *S. agalactiae.* * indicates significant difference (*p* < 0.05).

**Figure 11:**
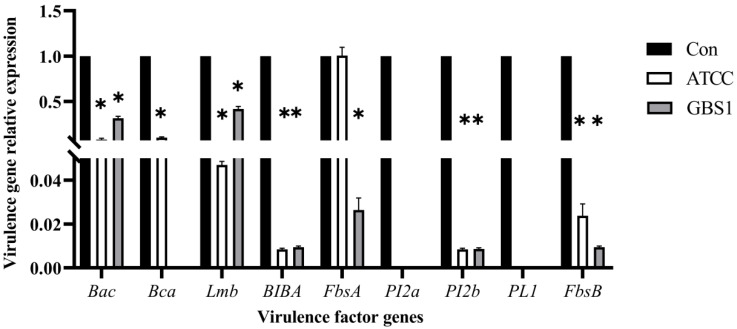
Effect of matrine on adhesion genes of *S. agalactiae*. * indicates significant difference (*p* < 0.05).

To further elucidate matrine’s mechanism of action, Kyoto Encyclopedia of Genes and Genomes (KEGG) enrichment analysis was conducted (Figure 9). The significantly differentially expressed genes in the ATCC strain were mainly associated with starch and sucrose metabolism, quorum sensing, and the bacterial secretion system. In contrast, differentially expressed genes in clinical isolates were primarily linked to nicotinate and nicotinamide metabolism, RNA degradation, and peptidoglycan biosynthesis. These findings provide insight into the molecular mechanisms by which matrine disrupts GBS virulence, supporting its potential as a natural antimicrobial alternative. Based on the transcriptome analysis, the key virulence genes with significant expression changes were verified by qRT-PCR. The results demonstrated that genes associated with invasion and immune evasion, including hemolysin (*CylE*), CAMP factor (*CAMP*), C5 complement-cleaving enzyme (*ScpB*), and capsular polysaccharide (*CpsA*), were significantly downregulated in both the ATCC strain and GBS clinical strains (*p* < 0.05). Additionally, genes related to adhesion and virulence factor expression, such as *CylE*, *CAMP*, *ScpB*, and *CpsA*, showed significant downregulation in both strain types (*p* < 0.05). Interestingly, the *CpsE* gene, which plays an important role in capsular polysaccharide production, was significantly upregulated (*p* < 0.05), which may be due to the adaptive strategy of the bacteria under drug pressure (*p* < 0.05) (Figure 10). Further analysis of genes associated with bacterial adhesion revealed that β-subunits (*Bac*), laminin-binding protein (*Lmb*), fimbrin (*PI-2b*), BIBA surface protein (*BIBA*), and fibrinogen-binding protein C (*FbsB*) were significantly downregulated in both the ATCC strain and GBS clinical strains (*p* < 0.05) (Figure 11). These findings suggest that matrine effectively disrupts key virulence pathways in GBS, potentially weakening its pathogenicity and adhesion ability.

## 4. Discussion

GBS plays a crucial role in mastitis development by modulating host immune responses and enhancing its survival in bovine mammary epithelial cells. Our results revealed that matrine significantly inhibited GBS survival in whole blood, suggesting its potential to impair immune evasion. While GBS is typically susceptible to penicillin, biofilm formation and intracellular persistence may reduce the efficacy of conventional antibiotics in chronic infections [18]. Our results suggest that matrine not only inhibits bacterial growth but also significantly reduces biofilm formation, which could complement existing antibiotic therapies. Neutrophil-mediated clearance and cytokine regulation play critical roles in bacterial persistence and disease progression [19]. Previous studies have shown that GBS can modulate immune factors such as TLR8 and complement factor C5 to suppress immune activation [20]. This immune suppression mechanism, facilitated by capsular polysaccharides, aligns with our findings that matrine effectively reduces capsule formation, thereby weakening GBS’s ability to evade host defenses [21].

Bacterial adhesion to host epithelial cells is a critical step in GBS colonization and infection. Our study demonstrated that matrine significantly reduced GBS adhesion to bovine mammary epithelial cells, a key factor in the early stages of mastitis. Streptococcal adhesion molecules, including *Lmb*, fibronectin-binding protein (*ScpA*), and fibrinogen-binding proteins (*Fbs*), are essential for host cell attachment and bacterial persistence [22]. Previous studies have suggested that the adhesion ability of GBS is largely determined by the expression of these virulence-associated genes rather than host-specific factors [23]. The observed reduction in adhesion following matrine treatment suggests that its inhibitory effect may be mediated through the downregulation of these key adhesion-related genes.

Capsular polysaccharides serve as a major virulence factor for GBS by enabling immune evasion. The capsule, consisting of terminal sialic acid (Sia) residues, mimics host structures and interferes with complement activation, preventing C3b deposition and impairing opsonophagocytosis [24]. The diversity of GBS capsule serotypes, with 10 known variations, plays a crucial role in its pathogenicity [25]. Moreover, Sia-binding to host Siglec receptors suppresses neutrophil oxidative burst, cytokine release, and phagocytosis, ultimately enhancing bacterial survival in the bloodstream [26]. Our study demonstrated that matrine significantly disrupted capsular integrity, which likely contributed to the reduced survival of GBS in whole blood. This aligns with previous findings that disrupting capsule biosynthesis weakens bacterial defenses, making them more susceptible to immune clearance.

The upregulation of *CpsE* under matrine treatment may represent a compensatory stress response by *S. agalactiae* to mitigate capsule disruption. Although *CpsE* encodes a key glycosyltransferase that initiates capsular polysaccharide biosynthesis [27], its transcriptional activation alone does not ensure the successful assembly of functional polysaccharide structures. We hypothesize that matrine may interfere with post-transcriptional or post-translational processes essential for capsule formation. For example, matrine or its metabolites could directly interact with the CpsE protein, impairing its catalytic function through allosteric inhibition, despite elevated mRNA expression levels [28]. Additionally, matrine-induced metabolic stress might uncouple gene expression from polysaccharide synthesis by depleting essential biosynthetic substrates or cofactors [29]. Furthermore, matrine’s reported membrane-targeting activity may disrupt the spatial organization of capsule export machinery, even in the presence of active biosynthesis genes, by affecting membrane microdomains critical for polysaccharide transport [30]. This observation is consistent with findings in *Streptococcus pneumoniae*, where antibiotic pressure upregulated capsule-related genes but ultimately impaired capsule export due to membrane damage [31]. Further investigations assessing CpsE protein levels, enzymatic activity, and capsule precursor availability under matrine exposure are warranted to elucidate the underlying mechanisms.

Transcriptomic analysis further elucidated matrine’s impact on GBS virulence regulation. The enrichment of pathways related to starch and sucrose metabolism, quorum sensing, and the bacterial secretion system suggests a potential inhibition of biofilm formation [32]. Quorum sensing (QS) is a crucial bacterial communication system that regulates biofilm development and coordinated gene expression [33]. Studies on *Pseudomonas aeruginosa* have shown that *SrpA*, a QS-related regulator, significantly promotes biofilm formation [34]. The inhibition of QS has been demonstrated to prevent biofilm production in *Staphylococcus aureus* through competitive antagonists like TrAIP-II [35,36]. Our findings suggest that matrine’s suppression of GBS biofilm formation may be linked to the interference with QS signaling, thereby reducing bacterial persistence.

The impairment of GBS biofilm development and capsule formation may also be linked to glycosaminoglycan metabolism. Glycosaminoglycans, linear polysaccharides composed of repeating disaccharide units, play essential roles in host cell adhesion and bacterial colonization [37]. GBS synthesizes hyaluronic acid-like glycosaminoglycans to evade immune detection. Our transcriptomic data showed significant enrichment in the glycosaminoglycan degradation pathway following matrine treatment, suggesting that matrine promotes glycosaminoglycan breakdown, thereby impairing capsule formation. Additionally, pathways related to peptidoglycan biosynthesis and ABC transporters, both essential for bacterial virulence, were also significantly affected [38]. The inhibition of these processes may further contribute to matrine’s antibacterial effects.

Gene expression analysis confirmed that matrine significantly downregulated key adhesion-related genes, including *Bac*, *Lmb*, *PI-2b*, *BIBA*, and *FbsB*. These genes encode proteins that mediate bacterial adherence, immune evasion, and host tissue colonization [39]. Fibrinogen-binding proteins (*FbsA* and *FbsB*) and *BIBA* surface proteins play crucial roles in epithelial and endothelial adhesion, facilitating immune escape [40]. Our findings suggest that matrine effectively suppresses these virulence-associated factors, thereby reducing GBS’s ability to adhere to host cells and establish infection.

## 5. Conclusions

The present study demonstrates that matrine effectively attenuates the virulence of *Streptococcus agalactiae* by inhibiting bacterial adhesion, reducing survival in whole blood, suppressing capsule and biofilm formation, and impairing persistence within established biofilms. Transcriptomic analysis further reveals that matrine exerts multi-target effects on key virulence-associated pathways, including those related to polysaccharide biosynthesis, adhesion, and membrane structure. To our knowledge, this is the first report to comprehensively elucidate the anti-virulence mechanisms of matrine against *S. agalactiae* at both phenotypic and transcriptomic levels.

Compared to conventional antibiotics, matrine offers several advantages, including its plant-derived origin, broad-spectrum antimicrobial activity, and lower potential for inducing antimicrobial resistance. However, the present study is limited to in vitro assays. Further in vivo investigations are needed to assess its pharmacokinetics, bioavailability, safety, and therapeutic efficacy in mastitis models. Overall, these findings not only expand our understanding of the antibacterial mechanisms of matrine but also highlight its promise as a natural alternative for the prevention and control of zoonotic mastitis, with potential applications in both veterinary and public health contexts.

## Data Availability

The original contributions presented in this study are included in the article/Supplementary Material. Further inquiries can be directed to the corresponding author.

## Supplementary Materials

The following supporting information can be downloaded at: https://www.mdpi.com/article/10.3390/microorganisms13061192/s1, Table S1: Primers sequences of virulence genes.

## Abbreviations

ATCC = ATCC 13813; Bac = β-subunits; BHI = brain–heart infusion; BIBA = BIBA surface protein; BMECs = Bovine mammary epithelial cells; BP = Biological Process; CAMP = CAMP factor; CC = Cellular Component; CLSI = Clinical and Laboratory Standards Institute; CLSM = confocal laser scanning microscopy; CMT = Mastitis Test; CpsA = capsular polysaccharide; CylE = hemolysin; Fbs = fibrinogen-binding proteins; FBS = fetal bovine serum; FbsB = fibrinogen-binding protein C; GBS = *Streptococcus agalactiae*; GO = Gene Ontology; KEGG = Kyoto Encyclopedia of Genes and Genomes; lmb = laminin-binding protein; MCHs = matrine–chitosan hydrogels; MF = Molecular Function; MOI = multiplicity of infection; PI = propidium iodide; PI-2b = fimbrin; qRT-PCR = Quantitative real-time PCR; QS = Quorum sensing; scpA = fibronectin-binding protein; ScpB = C5 complement-cleaving enzyme; Sia = sialic acid; SYTO9 = Syto 9 Green Fluorescent Nucleic Acid Stain; TSB = tryptic soy broth.
