# Supplementary material for: Matrine Attenuates Streptococcus agalactiae Virulence by Suppressing Capsular Polysaccharide Synthesis and Host Adhesion Pathways
Source: Microorganisms. 2025 May 23;13(6):1192. doi: 10.3390/microorganisms13061192 (PMC12195466; doi:10.3390/microorganisms13061192)
Supplement: Supplementary file 1 [file microorganisms-13-01192-s001.zip › microorganisms-3631789-supplementary.pdf]

Supplementary Table S1 Primers sequences of virulence genes

Genes	Primer sequences(5'-3')	Target fragment size/bp
SIP-F	GTATCAGCTCCAGCAGTTCCTGTG	93
SIP-R	TTGTGCTACCGGAACGCTCTTAAC	93
CAMP-R	CTGTCTCAGAGTTGGCACGC	156
CAMP-F	AAGCCCAGCAAATGGCTCAA	156
CPSE-F	AGAAGGTCTGACCACGAAGCATTG	98
CPSE-R	ATCCACATTGCACTAGGCGTCAC	98
16S-F	GCTCACCAAGGCGACGATACATAG	135
16S-R	GCGTTGCTCGGTCAGACTTCC	135
BIBA-F	AGCTAAGCAGTCAAGACGTTCTC	146
BIBA-R	TCAGTTGCGTGGCTTGTGTT	146
SCPB-F	AGCTGTCAACAATTGCCTTTTGCTT	80
SCPB-R	TATCAATGCTTTGTCAATGGCAACT	80
LMB-R	TGAATGCCTGCACCTGATTGGATC	110
LMB-F	ACGCAAGGCATGTCAGTTGTAACC	110
FBSA-F	AGCGTCGTCAACGTGATGCG	149
FBSA-R	ACGCTCTAGAACATTGCCTTGGC	149
FBSB-R	GCGAGGTCATTTCCGCAGTT	166
FBSB-F	ACTGCGCAAACTTCTGTCCA	166
PI-I-F	AGTGCTGATGCAACACCTGA	141
PI-I-R	TGTACGACGCTTCATCCCCT	141
PI-2a-F	AAGATGCTGAAGGTGGTGCTGTTC	194
PI-2a-R	GTCAATCCGTCACCATGGCAGAG	194
PI-2b-F	ACACAGGCGACTTATCAACAGGAG	105
PI-2b-R	AACCTGGCCTCGTTGGATCATTAG	105
CylE-F	AAGTCGTAGTGGACAGGCAATCAC	200
CylE-R	GGAGGTGACTACGCAATGATCGAC	200
Bac-F	CACCGAAGATTCCAGAGCTACCTC	175
Bac-R	GGAACACGCGGTGCTTCTGG	175
Bca-F	ACAATTCCAGGGAGTGCAGC	197
Bca-R	TGCAACTGAGAAACATCCCCA	197
CpsA-F	ACAACGCTTCACTGTCGAGTCAC	82
CpsA-R	AGCTCCTGGCATTGCATATGAAGG	82
HylB-F	GCTTCAACCGCAACTGCAACAG	139
HylB-R	CGCTTGTATGTGACCAGCTTCCAG	139
